# CHILDHOOD SOCIOECONOMIC STATUS GRADIENTS IN FUNCTIONAL LIMITATIONS: THE ROLE OF MACRO-ECONOMIC CONTEXT

**DOI:** 10.1093/geroni/igad104.3222

**Published:** 2023-12-21

**Authors:** Thomas Fuller-Rowell, Samia Sultana, Amy Hudson, Eric Kim, Carol Ryff

**Affiliations:** Auburn University, Auburn, Alabama, United States; Auburn University, Auburn, Alabama, United States; Auburn University, Auburn, Alabama, United States; University of British Columbia, Vancouver, British Columbia, Canada; University of Wisconsin--Madison, Madison, Wisconsin, United States

## Abstract

A large number of studies have documented an association between childhood socioeconomic status (SES) and adult health or illness. However, relatively little is known about how this SES-health gradient varies across macro-economic contexts. The current study examined state-level mean income as a moderator of the association between the education level of a person’s parents and functional limitations in the United States (N = 10,685, Mean age = 47.7, SD = 13.6, 51% female, 86% White; 6% Black; 8% other or missing). Data were from an economic time series database (1917-2018) and the Midlife in the United States Study (MIDUS). Time series data were merged with MIDUS data (1995 core sample and 2012 refresher) by state of residence and year to determine exposure to state economic characteristics during three periods of the life span: prime working years (ages 30-55), early adulthood (20-25) and childhood (0-15). Results indicated that the SES-functional limitations gradient was attenuated for those who lived in a state with a higher mean income from ages 30-55, 20-25, and 0-15 (p’s < .05). The moderating role of state-level economic exposures between ages 30 and 55 (but not at earlier life stages) differed by gender such that the attenuation of the SES-functional limitations gradient in more affluent states was more strongly evident among females than males. Findings suggest that the role of childhood SES in later life health varies across state-level economic contexts with notable differences by gender and age group. Future directions and policy implications are discussed.

